# Comprehensive analysis of the papillary thyroid carcinoma identifies CSGALNACT1 as a proliferation driver and prognostic biomarker

**DOI:** 10.3389/fcell.2025.1638348

**Published:** 2025-08-29

**Authors:** Yu Zhao, Juan Zhang, Yong Yao, Linna Yu

**Affiliations:** ^1^ People’s Hospital of Qianxinan Prefecture, Xingyi, Guizhou, China; ^2^ Department of Pharmaceutics, Key Laboratory of Drug Quality Control and Pharmacovigilance (Ministry of Education), State Key Laboratory of Natural Medicines, NMPA Key Laboratory for Research and Evaluation of Pharmaceutical Preparations and Excipients, China Pharmaceutical University, Nanjing, China

**Keywords:** papillary thyroid carcinoma, ScRNA-seq, bulk RNA-seq, immune infiltration, cell proliferation

## Abstract

Papillary thyroid carcinoma (PTC) is the most prevalent form of thyroid cancer, yet its cellular heterogeneity and prognostic determinants remain poorly defined. Here, we integrate two single-cell RNA sequencing datasets comprising 20 human thyroid samples to construct a high-resolution cellular atlas of PTC. We identify 29 distinct cellular subpopulations and delineate their composition, dynamics, and interactions in healthy versus tumor tissues. Notably, epithelial and monocyte populations were markedly expanded in PTC, whereas adaptive immune subsets such as B and T cells were diminished. Cell–cell communication analysis revealed enhanced intercellular signaling in the tumor microenvironment, with epithelial and endothelial cells receiving the strongest inputs. Among monocyte-specific transcriptional signatures, we identified 65 prognostic genes via univariate Cox analysis. A LASSO-derived 14-gene risk score robustly stratified patient outcomes, with CSGALNACT1 emerging as a key epithelial-specific, independent prognostic gene. Pseudotime analysis further supported its role in epithelial cell differentiation. Functional validation demonstrated that CSGALNACT1 promotes proliferation in PTC cell lines, suggesting a potential oncogenic function. Immune deconvolution across risk groups revealed substantial divergence in innate and adaptive immune infiltration, indicating a close interplay between tumor-intrinsic transcriptional programs and immune microenvironment remodeling. Collectively, our study provides a comprehensive single-cell framework for PTC, identifies a clinically relevant risk model, and highlights CSGALNACT1 as a potential therapeutic target.

## 1 Introduction

Papillary thyroid carcinoma (PTC) is the most common subtype of thyroid malignancy, characterized by a generally indolent course but marked heterogeneity in clinical behavior and risk of recurrence (Boucai et al., 2024). Although most patients respond well to surgery and radioactive iodine therapy, a small percentage of patients develop aggressive disease characterized by local invasion or distant metastasis (Wang et al., 2024a). Despite advances in genomics and therapeutic drug development, the molecular mechanisms driving PTC tumor progression and heterogeneity remain incompletely elucidated (Song and Park, 2019; Sun and Liao, 2024).

With the development of omics assays and their reduced cost, more and more tumor genetic programs and tumor microenvironments have been revealed (Galassi et al., 2024; Yang et al., 2023). For PTC-related studies, bulk transcriptome analyses have obtained valuable prognostic features; however, they lack cellular resolution and may obscure the role of cell type-specific drivers (Amanullah et al., 2023; Zhao et al., 2024). Single-cell RNA sequencing (scRNA-seq) provides a powerful strategy to resolve tumor heterogeneity, track lineage trajectories, and identify cell type-specific and functionally relevant molecular markers (Yan et al., 2024; Hou et al., 2024). Therefore, combining the two and validating them through functional experiments can provide favorable support for the study of molecular mechanisms and prognostic markers of PTC.

This study integrated bulk and single-cell transcriptome data from two independent papillary carcinoma (PTC) cohorts, constructed a robust prognostic model, and identified key epithelial-specific regulators of tumor progression. Among the six independent prognostic genes, we focused on CSGALNACT1, which showed high specificity in epithelial cells and was highly consistent with pseudo-time-derived differentiation trajectories. Functional analysis showed that CSGALNACT1 promoted tumor cell proliferation, suggesting that it may have a previously unrecognized oncogenic role.

## 2 Methods

### 2.1 Data sources

**FIGURE 1:**
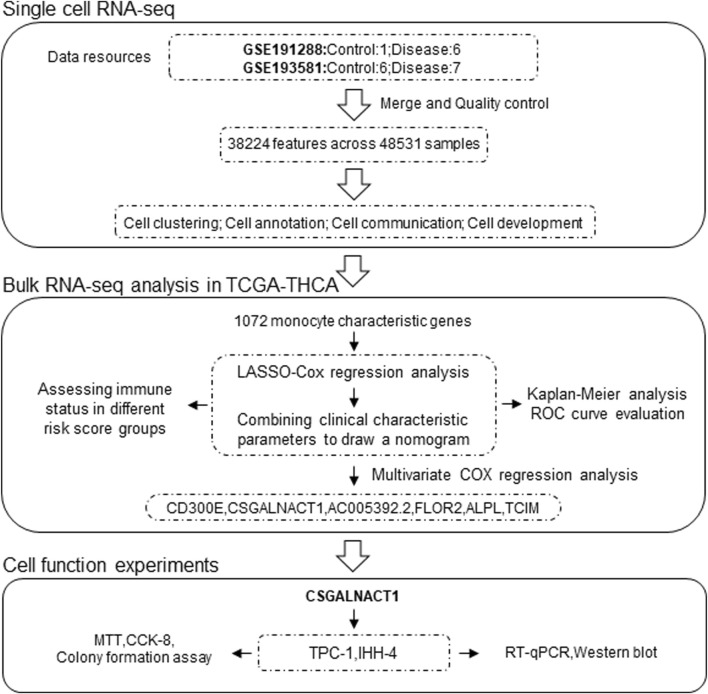
Working mode.

The study design and analysis workflow are illustrated in Figure 1. Single-cell RNA sequencing (scRNA-seq) data were obtained by integrating two independent cohorts: GSE191288 (1 control, 6 tumor) and GSE193581 (6 control, 7 tumor), resulting in a total of 48,531 cells and 38,224 gene expression features after quality control and data integration (Jin et al., 2021; Lu et al., 2023). Bulk RNA-seq data and corresponding clinical information were retrieved from the TCGA-THCA cohort.

### 2.2 Single-cell transcriptomic analysis

Quality control, normalization, dimensionality reduction, and clustering of the scRNA-seq data were performed using the Seurat package. Principal component analysis (PCA) and t-distributed stochastic neighbor embedding (t-SNE) were applied for dimensionality reduction (Satija et al., 2015). Differentially expressed genes (DEGs) between groups were identified using the “FindMarkers” function in Seurat with default parameters and statistical significance assessed by Wilcoxon rank-sum test (adjusted P < 0.05). Cell types were annotated using the SingleR package based on reference transcriptomic profiles to identify cell-type-specific marker genes (Aran et al., 2019).

### 2.3 Cell–cell communication and pseudotime trajectory analysis

Cell–cell communication networks were inferred using the CellChat package by analyzing ligand–receptor interactions among different cell subsets (Jin et al., 2021). Pseudotime trajectory analysis was conducted using Monocle 3, an unsupervised algorithm that reconstructs developmental trajectories and orders cells along differentiation processes relevant to biological progression (Cao et al., 2019).

### 2.4 Construction of the prognostic risk score

Multivariate Cox regression analysis was performed using the survival R package to identify independent prognostic factors. A prognostic risk score model was then constructed based on LASSO-Cox regression using the TCGA-THCA dataset. To enhance predictive accuracy and clinical utility, a nomogram was generated by integrating the risk score with clinical parameters. Patients were stratified into high- and low-risk groups according to the median risk score. Kaplan–Meier survival analysis was used to evaluate overall survival (OS), and time-dependent receiver operating characteristic (ROC) curves were employed to assess the predictive performance of the model.

### 2.5 Assessment of immune cell infiltration

Immune cell infiltration and immune-related scores in the TCGA-THCA cohort were estimated using six computational algorithms: ESTIMATE, TIMER, MCP-counter, CIBERSORT, EPIC, and quanTIseq (Li et al., 2017; Chen et al., 2018; Racle and Gfeller, 2020; Finotello et al., 2019). The association between immune scores and the risk score was assessed using Spearman’s rank correlation. Differences in the proportions of immune cell subtypes between high- and low-risk groups were evaluated using the Wilcoxon rank-sum test.

### 2.6 Plasmid construction and cell culture/transfection

The full-length coding sequence of human CSGALNACT1 (NM_001130518.2) was amplified by PCR and cloned into the CMV-FLAG expression vector, as previously described^10^. The human papillary thyroid carcinoma cell lines IHH-4 and TPC-1 were kindly provided by Prof. Yan Yang (Zunyi Medical University, China). Cells were maintained in Dulbecco’s Modified Eagle Medium (DMEM; Gibco, Cat# 11965092) supplemented with 10% fetal bovine serum (FBS; Gibco, Cat# 10099141) and 1% penicillin–streptomycin (Gibco, Cat# 15140122) at 37 °C in a humidified incubator with 5% CO_2_ (Zeng et al., 2018).

For overexpression experiments, cells were transfected with either empty CMV-FLAG vector or FLAG-tagged CSGALNACT1 using Neofect™ DNA transfection reagent (Neofect Biotech, Beijing, China) according to the manufacturer’s protocol. For gene knockdown, three small interfering RNAs (siRNAs) targeting CSGALNACT1 were designed and synthesized (GenePharma, China). The target sequences were as follows:

si-CSGALNACT1#1-F: 5′-GGUAGUUUAUGAAAUUUAAUU-3′,

si-CSGALNACT1#1-R: 5′-UUAAAUUUCAUAAACUACCAG-3′

si-CSGALNACT1#2-F: 5′-GAUUUGUACUGGUAGUUUAUG-3′,

si-CSGALNACT1#2-R: 5′-UAAACUACCAGUACAAAUCAA-3′

si-CSGALNACT1#3-F: 5′-GGAAUGGUUUGUACUAAUACA-3′,

si-CSGALNACT1#3-R: 5′-UAUUAGUACAAACCAUUCCUU-3′

### 2.7 Western blotting and quantitative real-time PCR

Forty-eight hours after transfection with control or FLAG–CSGALNACT1 plasmids (Neofect™ DNA transfection reagent, Neofect Biotech, China), cells were lysed in 1% SDS buffer supplemented with protease and phosphatase inhibitors (Apexbio, Cat# A601668). Protein concentrations were quantified using a BCA assay (Thermo Scientific, Cat# 23227). Equal amounts of total protein were separated by SDS–PAGE and transferred to PVDF membranes (Millipore, Cat# IPVH00010). Membranes were probed with primary antibodies against CSGALNACT1 (1:1,000; Novus Biologicals, Cat# NBP2-59219), FLAG (1:5,000; Proteintech, Cat# 20543-1-AP), and β-actin (1:5,000; Proteintech, Cat# 66009-1-Ig), followed by incubation with HRP-conjugated secondary antibodies (CWBIO, CW0103S (Anti-Rabbit), CW0102S (Anti-Mouse), 1:3000). Signal detection was performed using an enhanced chemiluminescence (ECL) reagent (Smart-Lifesciences, Cat# SL-ECL002).

For quantitative real-time PCR (qRT-PCR), total RNA was extracted using Trizol reagent (Invitrogen, Cat# 15596026) and reverse-transcribed with a cDNA synthesis kit (Vazyme, Cat# R223-01). Expression levels were normalized to 18S rRNA using the ΔΔCt method. qPCR was performed using gene-specific primers and SYBR Green Master Mix (Takara, Cat# RR820A) and quantified by CFX96 real-time PCR detection (CFX96; Bio-Rad, United States) systems. Primer sequences were as follows:

CSGALNACT1-F: 5′-TGGACAAGGCAGAGGTGAAT-3′

CSGALNACT1-R: 5′-TCTCTGCAGGACTGTTCAGG-3′

18S-F: 5′-GTAACCCGTTGAACCCCATT-3′

18S-R: 5′-CCATCCAATCGGTAGTAGCG-3′

### 2.8 Cell proliferation assay

Cell proliferation was evaluated using MTT (Sigma-Aldrich, Cat# M2128), CCK-8 (Dojindo Laboratories, Cat# CK04), and colony formation assays. For the MTT assay, IHH-4, TPC-1 and B-CPAP cells were counted using a Neubauer hemocytometer and seeded into 96-well plates at a density of 2,000 cells per well. After 48 h of incubation, 20 μL of MTT solution (5 mg/mL in PBS) was added to each well and incubated for an additional 4 h at 37 °C in a humidified incubator with 5% CO_2_. Subsequently, 150 μL of dimethyl sulfoxide (DMSO; Sigma-Aldrich, Cat# D2650) was added to each well to dissolve the formazan crystals. The plates were gently shaken for 10 min, and absorbance was measured at 490 nm using a microplate reader (EnSpire 2300, PerkinElmer, United States). CCK-8 and clonogenic assays were performed as described previously (Zhao et al., 2025).

### 2.9 Statistical analysis

Group differences were assessed using the Wilcoxon test, and associations were analyzed using Spearman rank correlation. All statistical tests were two-sided, with significance defined at P < 0.05. Statistical analyses were performed using R software (version 4.2.2).

## 3 Result

### 3.1 Single-cell characteristics of papillary thyroid carcinoma

**FIGURE 2:**
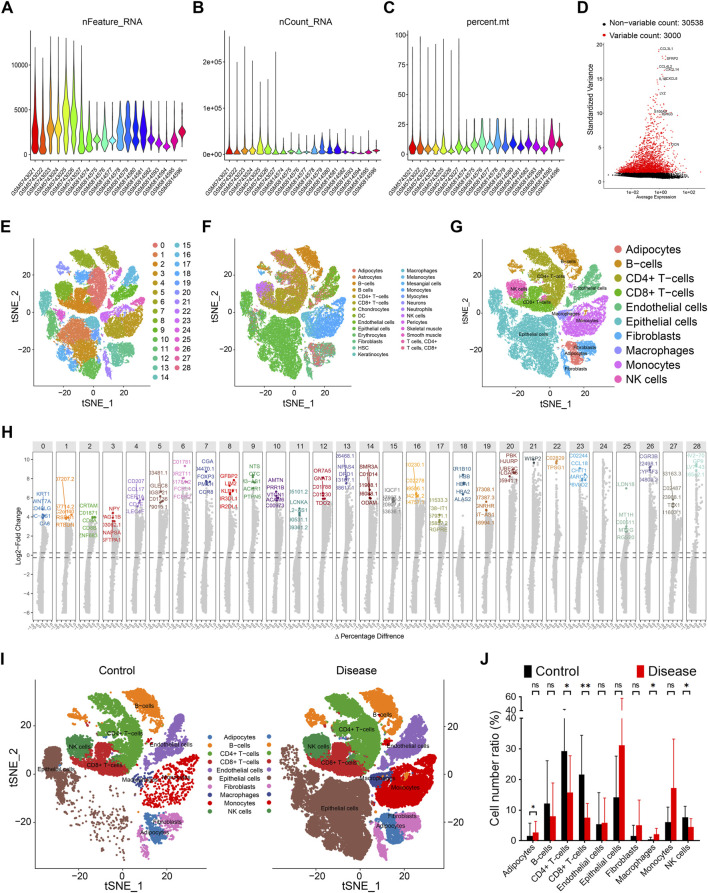
Single cell features of papillary thyroid cancer. **(A)** Gene quantity features of data quality control. **(B)** Cell distribution of data quality control. **(C)** Gene content of silience in data quality control. **(D)** Top 3000 highly variable genes of all samples. **(E)** 29 cell clusters are represented by tSNE diagram, and different colors represent different cell subpopulations. **(F)** Automatic annotation of 29 cell clusters. **(G)** Manual correction of cell annotation into 10 cell clusters, represented by tSNE diagram. **(H)** Top 5 characteristic genes of 29 cell subpopulations, ranked by change fold. **(I)** Distribution of 10 cell clusters in the control group, represented by tSNE diagram. **(J)** Distribution of 10 cell clusters in the disease group, represented by tSNE diagram.

By integrating datasets GSE191288 and GSE193581, we obtained a total of 7 control and 13 papillary thyroid carcinoma (PTC) samples. Following rigorous quality control, 48,531 cells with 38,224 transcriptomic features were retained for downstream analyses (Figures 2A–C). Using the Seurat package, we identified 3,000 highly variable genes across the dataset, which were subsequently used for cell clustering and annotation (Figure 2D). At an optimized resolution, 29 distinct cellular clusters were identified and automatically annotated using the SingleR algorithm (Figures 2E,F). Based on canonical marker genes and prior literature, these clusters were further classified into 10 major cell types: adipocytes, B cells, CD4^+^ T cells, CD8^+^ T cells, epithelial cells, endothelial cells, fibroblasts, macrophages, monocytes, and natural killer (NK) cells (Figures 2G,H). Notably, compared with controls, PTC samples exhibited a marked increase in macrophages, accompanied by a reduction in other cells, including CD4^+^ T cells, CD8^+^ T cells, and NK cells (Figures 2I,J).

### 3.2 Enhanced cell–cell communication in papillary thyroid carcinoma

**FIGURE 3:**
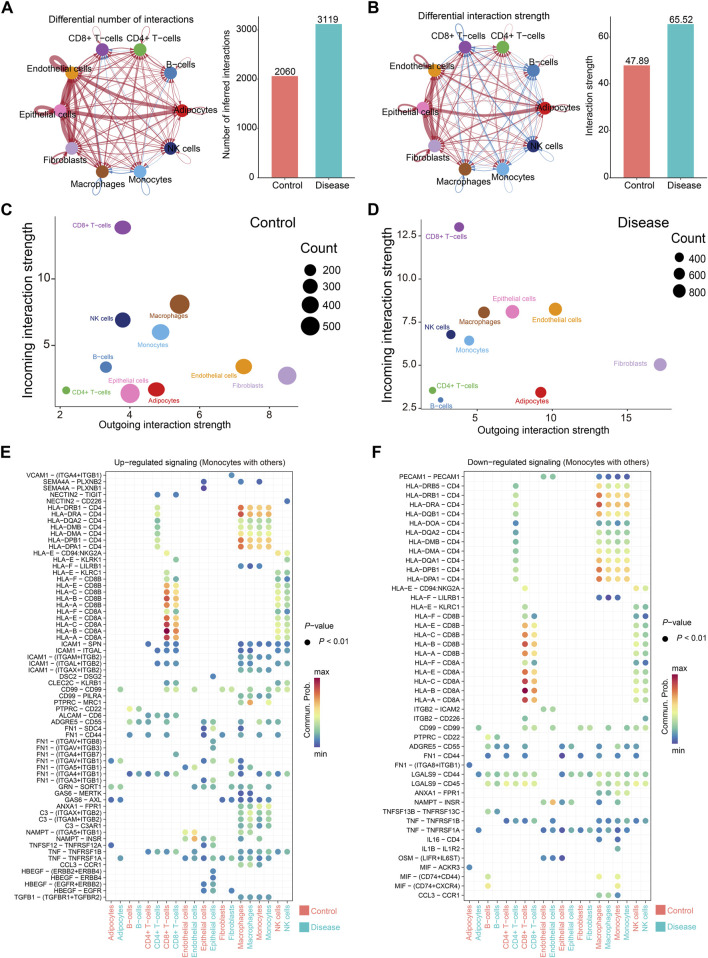
Cell communication in papillary thyroid cancer. **(A,B)** Cell communication between all samples. On the left are cell communication associations between different cells. The thickness of the line represents the number of cell communications **(A)** or intensity **(B)**; on the right are comparisons of the number of communications **(A)** or intensity **(B)** between the control group and the disease group. **(C,D)** The total number and intensity of communications between specific cell subsets and other cells in the control group **(C)** or the disease group **(D)**. The size of the circle represents the number of communications, the horizontal axis represents the signal output, and the vertical axis represents the signal input. **(E,F)** Comparison of changes in communication signals between monocytes and other cells, arranged in the order of upregulation **(E)** or downregulation **(F)**. The color of the circle represents the communication intensity.

Cell–cell communication patterns in control and papillary thyroid carcinoma (PTC) samples were inferred using the CellChat R package. Overall, strong intercellular signaling was observed among endothelial cells, epithelial cells, fibroblasts, and adipocytes in both control and disease groups (Figures 3A,B). Notably, the total number and strength of cell–cell interactions were markedly increased in the PTC group compared to controls (Figures 3A,B). Specifically, endothelial cells and fibroblasts exhibited the strongest outgoing signals, whereas CD8^+^ T cells received the most robust incoming signals (Figures 3C,D). In contrast, CD4^+^ T cells and B cells showed relatively weak signaling activities, both in terms of sending and receiving signals (Figures 3C,D). Of particular interest, the incoming signals to epithelial and endothelial cells were significantly enhanced in the PTC group (Figures 3C,D).

Monocytes displayed diverse changes in communication patterns depending on the ligand–receptor pairs and interacting cell types (Figures 3E,F). Among these, the most prominent interactions occurred between monocytes and CD8^+^ T cells, primarily mediated by CD8A and HLA family molecules (Figures 3E,F). This was followed by strong signaling between monocytes and macrophages, as well as autocrine communication among monocytes, with CD4–HLA interactions being the dominant signaling route (Figures 3E,F).

### 3.3 Construction of a monocyte-derived prognostic risk score

**FIGURE 4:**
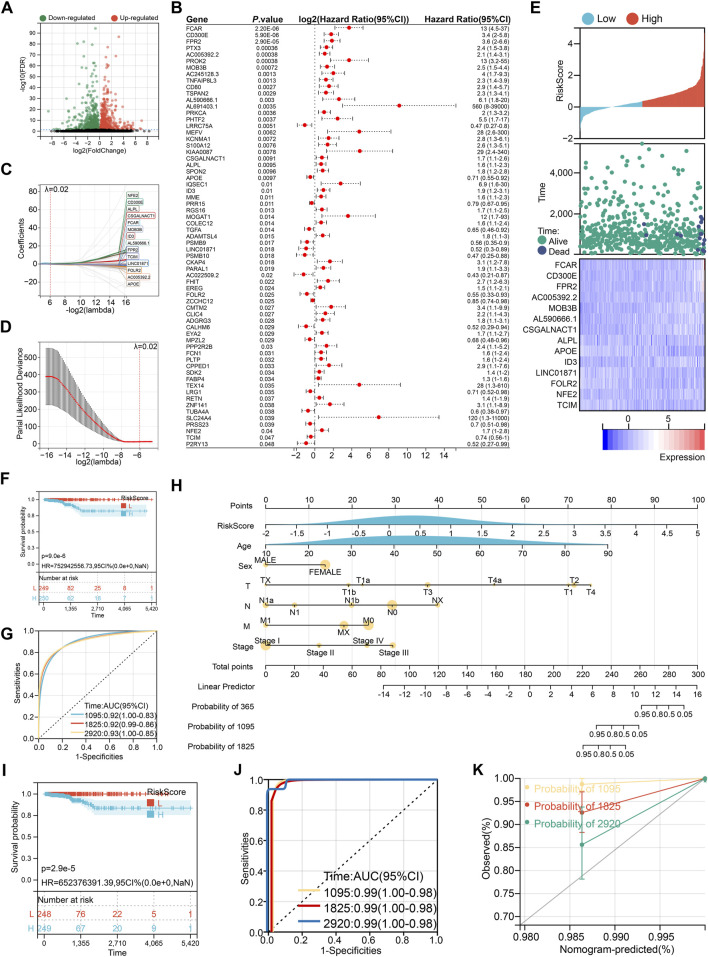
Construction of prognostic risk score. **(A)** Volcano plot of monocyte characteristic genes. **(B)** Univariate Cox regression to screen prognostic characteristic genes. **(C)** 14 genes selected by LASSO-Cox regression algorithm at λ = 0.02. **(D)** The process of repeated sampling test in LASSO-Cox regression algorithm, and finally the appropriate coefficient was obtained at λ = 0.02. **(E)** The association between risk score wind resistance, patient survival status and 14 gene expression. **(F)** Kaplan-Meier survival analysis based on risk score grouping. **(G)** ROC curve to evaluate the prognostic prediction efficiency of risk score. **(H)** Nomogram drawn by risk score combined with clinical characteristics. **(I)** Kaplan-Meier survival analysis based on nomogram group. **(J)** ROC curve to evaluate the prognostic prediction efficiency of nomogram. **(K)** Calibration curve of nomogram.

A total of 503 highly variable genes were extracted from the monocyte cluster based on criteria of log_2_ fold change >1 and false discovery rate (FDR) < 0.05. These genes were subjected to univariate Cox regression analysis, yielding 65 genes significantly associated with patient prognosis (Figures 4A,B). To construct an optimal prognostic model, we applied the LASSO-Cox regression algorithm (λ = 0.02), which identified a 14-gene signature used to calculate a monocyte-derived risk score (Figures 4C,D).

$$\begin{array}{ll} \text{RiskScore} & =0.8998*\text{FCAR}+0.5334*\text{CD}300E+0.3896*\text{FPR}2\\ & \;+0.4386*\text{AC}005392.2+0.1090*\text{MOB}3B\\ & \;+0.5997*\text{AL}590666.1+0.0989*\text{CSGALNACT}1\\ & \;+0.1551*\text{ALPL}-0.03101*\text{APOE}+0.0345*\text{ID}3\\ & \;-0.0443*\text{LINC}01871-0.2477*\text{FOLR}2\\ & \;+0.0347*\text{NFE}2-0.1597*\text{TCIM} \end{array}$$

This risk score, along with the expression levels of its component genes, was strongly correlated with overall survival (Figure 4E). Patients stratified into the high-risk group exhibited significantly poorer survival outcomes, with a lower median survival time (Figure 4F). The area under the curve (AUC) values for 3-, 5-, and 8-year survival prediction were 0.92, 0.92, and 0.93, respectively, indicating high diagnostic accuracy (Figure 4G). To enhance clinical applicability, we integrated the risk score with key clinical variables (age, sex, TNM stage, and tumor stage) to construct a nomogram (Figure 4H), which further improved prognostic discrimination (Figures 4I,J). However, it is noteworthy that the nomogram tended to underestimate patient survival, particularly at 3, 5, and 8 years, suggesting a conservative bias in survival prediction (Figure 4K).

### 3.4 Immune landscape differences between risk groups

**FIGURE 5:**
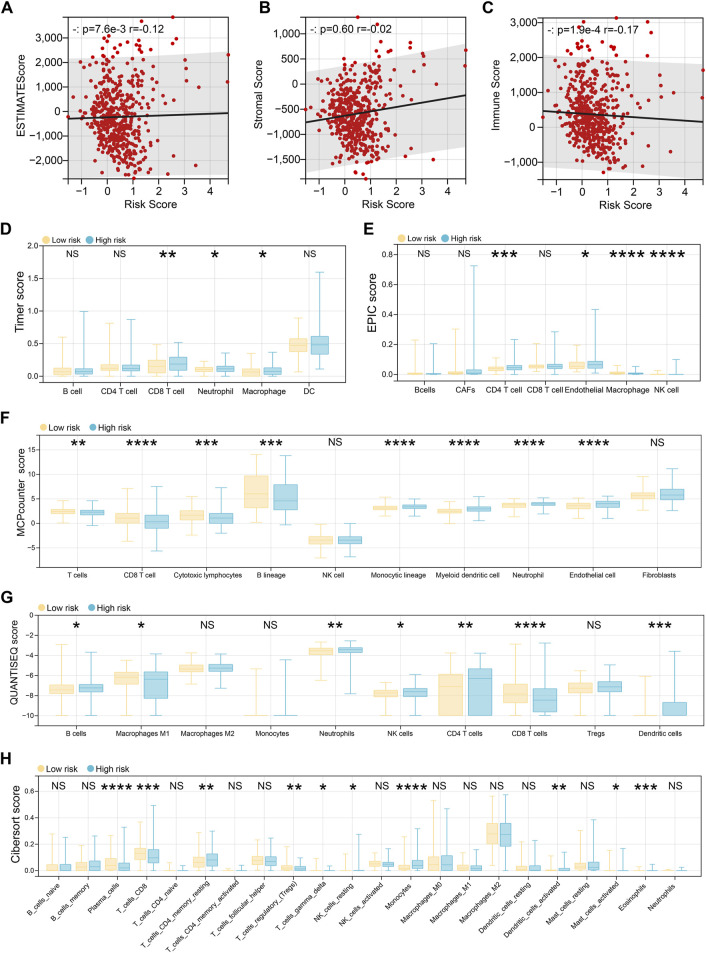
Immune landscape differences between risk groups. **(A–C)** The ESTIMATE algorithm evaluates the patient’s estimate score **(A)**, stromal score **(B)**, and immune score **(C)**, and then the spearman method is used to evaluate the correlation with the risk score. **(D)** The Timer algorithm calculates the patient’s immune cell infiltration, and then performs a difference test based on the risk score group. **(E)** The EPIC algorithm calculates the patient’s immune cell infiltration, and then performs a difference test based on the risk score group. **(F)** The MCPcounter algorithm calculates the patient’s immune cell infiltration, and then performs a difference test based on the risk score group. **(G)** The QUANTISEQ algorithm calculates the patient’s immune cell infiltration, and then performs a difference test based on the risk score group. **(H)** The CIBERSORT algorithm calculates the patient’s immune cell infiltration, and then performs a difference test based on the risk score group.

To investigate immune status across risk groups, six computational algorithms were applied to evaluate overall immune scores and immune cell infiltration patterns. While the three immune scores derived from the ESTIMATE algorithm showed no significant correlation with the risk score, other methods revealed marked differences in immune cell infiltration between high- and low-risk groups (Figures 5A–C). Specifically, the TIMER algorithm indicated that high-risk patients exhibited elevated infiltration of CD8^+^ T cells, neutrophils, and macrophages (Figure 5D). The EPIC algorithm further suggested increased infiltration of CD4^+^ T cells, endothelial cells, macrophages, and natural killer (NK) cells in the high-risk group (Figure 5E). According to the MCP-counter analysis, significant differences were observed in 8 of the 10 assessed immune cell types, with the exception of NK cells and fibroblasts (Figure 5F). These differences included higher levels of T cells, macrophages, and neutrophils in the high-risk group. Similarly, the quanTIseq algorithm identified differential infiltration of seven immune cell types, including B cells, M1 macrophages, neutrophils, and NK cells (Figure 5G). Finally, CIBERSORT analysis, which evaluates 22 immune cell populations, revealed additional differences, including altered infiltration of plasma cells, dendritic cells, and eosinophils (Figure 5H).

### 3.5 Epithelial-specific expression of the independent prognostic gene CSGALNACT1

**FIGURE 6:**
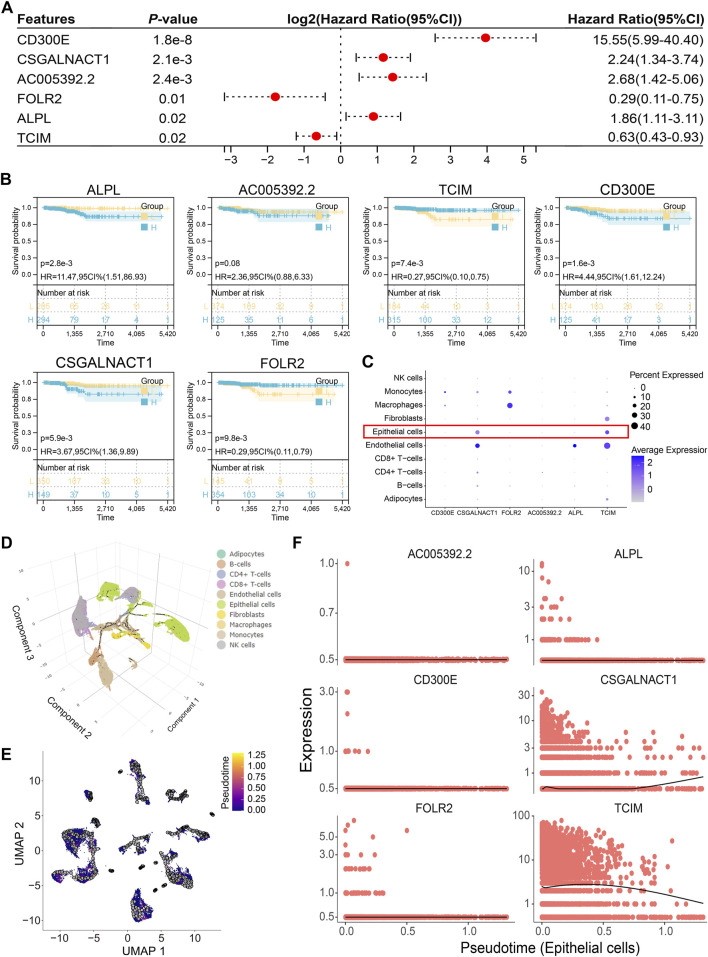
Epithelial-specific expression of the independent prognostic gene CSGALNACT1. **(A)** Multivariate COX regression analysis of 14 risk score genes. **(B)** Kaplan-Meier survival analysis of 6 independent prognostic genes. **(C)** Cell subpopulation expression characteristics of 6 independent prognostic genes in single-cell datasets. **(D)** Single-cell pseudo-time analysis of all samples. **(E)** Pseudo-time analysis of epithelial cells. **(F)** Fitting relationship between epithelial cell development trajectory and transcription levels of 6 genes.

Multivariate Cox regression analysis of the 14 genes comprising the risk score identified six independent prognostic factors: CD300E, CSGALNACT1, AC005392.2, FOLR2, ALPL, and TCIM (Figure 6A). Among these, all but AC005392.2 demonstrated prognostic relevance based on transcriptional expression levels (Figure 6B). Given that papillary thyroid carcinoma originates from epithelial cells, we focused on genes with epithelial-specific expression. Single-cell transcriptomic analysis revealed that CSGALNACT1 and TCIM were predominantly expressed in epithelial cells, in contrast to the other candidate genes (Figure 6C). To further characterize their dynamic expression patterns, pseudotime trajectory analysis was performed on all samples (Figure 6D), followed by developmental trajectory modeling specifically within epithelial cells (Figure 6E). Notably, among the six prognostic genes, CSGALNACT1 and TCIM showed expression trends that closely aligned with the inferred epithelial cell differentiation trajectory (Figure 6F).

### 3.6 CSGALNACT1 promotes cell proliferation in papillary thyroid cancer

**FIGURE 7:**
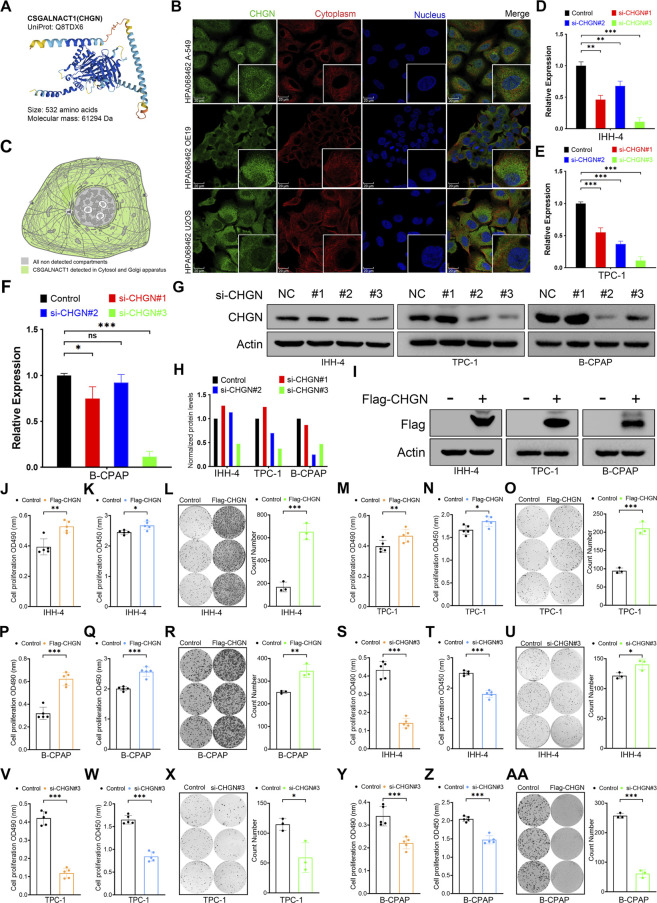
CSGALNACT1 promotes cell proliferation in papillary thyroid cancer. **(A)** Protein structure of CSGALNACT1. **(B)** Cellular sublocalization of CSGALNACT1, cell immunofluorescence staining from HPA database. **(C)** Cellular sublocalization pattern of CSGALNACT1. **(D–F)** Real-time fluorescence quantitative PCR to verify the inhibition efficiency of siRNA in IHH-4 **(D)**, TPC-1 **(E)**, and B-CPAP **(F)** cell line. **(G,H)** Immunoblotting to verify the inhibition efficiency of siRNA in IHH-4, TPC-1, and B-CPAP cell line **(G)**, and quantitative analysis was performed **(H)**. **(I)** Immunoblotting to verify the overexpression of Flag-CSGALNACT1 in IHH-4, TPC-1, and B-CPAP cell line. **(J–L)** MTT **(J)**, CCK8 **(K)**, and clone formation assay **(L)** to detect the proliferation ability of IHH-4 cells in the control group and the group overexpressing CSGALNACT1. **(M–O)** MTT **(M)**, CCK8 **(N)**, and clone formation assay **(O)** to detect the proliferation ability of TPC1 cells in the control group and the group overexpressing CSGALNACT1. **(P–R)** MTT **(P)**, CCK8 **(Q)**, and clone formation assay **(R)** to detect the proliferation ability of B-CPAP cells in the control group and the group overexpressing CSGALNACT1. **(S–U)** MTT **(S)**, CCK8 **(T)**, and clone formation assay **(U)** were used to detect the proliferation ability of IHH-4 cells in the control group and the si-CSGALNACT1#3 group. **(V–X)** MTT **(V)**, CCK8 **(W)**, and clone formation assay **(X)** were used to detect the proliferation ability of TPC1 cells in the control group and the si-CSGALNACT1#3 group. **(Y-AA)** MTT **(Y)**, CCK8 **(Z)**, and clone formation assay **(AA)** were used to detect the proliferation ability of B-CPAP cells in the control group and the si-CSGALNACT1#3 group.

CSGALNACT1 (also known as CHGN) encodes a 532-amino acid protein with a predicted molecular weight of 61.3 kDa (Figure 7A). Immunofluorescence data from the Human Protein Atlas (HPA) revealed that CHGN is primarily localized to the cytoplasm and Golgi apparatus (Figures 7B,C). To investigate its role in cell proliferation, we performed both knockdown and overexpression experiments. The efficiency of CHGN silencing was validated by qRT-PCR and Western blotting, with si-CHGN#3 exhibiting the most effective knockdown and thus selected for subsequent experiments (Figures 7D–H). Overexpression of CHGN using a Flag-tagged construct was confirmed in IHH-4 cells via Western blotting (Figure 7I). Functional assays, including MTT, CCK-8, and colony formation, demonstrated that CHGN overexpression significantly promoted proliferation in IHH-4 and TPC-1 cells (Figures 7J–O), consistent with results observed in B-CPAP cells (Figures 7P–R). Conversely, CHGN knockdown markedly suppressed cell proliferation in both IHH-4, TPC-1 and B-CPAP cells, as validated across all three proliferation assays (Figures 7S–AA).

## 4 Discussion

In this study, we systematically integrated single-cell and bulk transcriptomic data to construct a prognostic model for papillary thyroid carcinoma (PTC) and identified CSGALNACT1 as a novel epithelial-specific oncogenic regulator. Single-cell RNA sequencing revealed that CSGALNACT1 is predominantly expressed in epithelial tumor cells and closely associated with epithelial differentiation trajectories, suggesting a lineage-specific role in PTC progression. Functional assays confirmed that CSGALNACT1 promotes cell proliferation *in vitro*, establishing it as both a prognostic biomarker and a potential therapeutic target.

To ensure robustness and reproducibility, we analyzed two independent single-cell PTC datasets, a strategy increasingly adopted in cancer single-cell transcriptomic studies (Zhang et al., 2025). Cell–cell communication analysis highlighted monocyte-associated signaling—particularly involving CD8, CD4, and HLA molecules—as the most perturbed in PTC. These findings are consistent with previous reports linking monocyte-attracting chemokines to lymph node metastasis and tumor recurrence in PTC (Tanaka et al., 2009). Notably, CD8^+^ T cells have also been proposed as prognostic markers for PTC recurrence (Chen et al., 2022), potentially through the activation of the DPP4–IL13–IL13RA2 axis (Jing et al., 2025), further underscoring the immune microenvironment’s role in PTC biology.

Our risk score model, derived from LASSO-Cox regression and integrated with clinical parameters into a nomogram, achieved excellent predictive performance (AUC >0.90). While this performance is comparable to recent studies (Zhao et al., 2024; Liang et al., 2024), it may be partly attributable to the generally indolent nature of PTC and the use of a single-cohort design. Notably, calibration curves indicate a conservative bias in survival prediction, wherein the model tends to underestimate patient survival. Mathematically, this bias may arise from class imbalance in outcome events within the training cohort. Clinically, such bias could lead to overtreatment, as the model overestimates mortality risk. However, current clinical consensus is shifting away from aggressive interventions toward active surveillance for low-risk thyroid cancers. Evidence suggests that active surveillance does not compromise prognosis in these patients (Levyn et al., 2024; Walgama et al., 2020). Therefore, before clinical application, the model requires further refinement incorporating additional modalities such as proteomic data, non-coding RNA profiles, and radiomic features (Wang et al., 2024b; Zhu et al., 2024; Cao et al., 2024).

Importantly, immune infiltration analysis revealed that high-risk patients exhibited elevated infiltration of immunosuppressive cells and higher stromal scores, suggesting an immune-evasive tumor microenvironment. These results suggest that intrinsic tumor features, such as CSGALNACT1 overexpression, may contribute not only to increased proliferation but also to immune remodeling. This dual role provides a compelling rationale for combinatorial therapies targeting both tumor-intrinsic pathways and the surrounding immune contexture (Wu et al., 2024; Li et al., 2025).

CSGALNACT1 encodes a critical glycosyltransferase previously implicated in neuronal and skeletal development (Meyer et al., 2019; Igarashi et al., 2018). Our study functionally validated its pro-proliferative role in PTC, as demonstrated through gain- and loss-of-function experiments in two independent cell lines. Subcellular localization analysis showed that the CSGALNACT1 protein product, CHGN, is mainly distributed in the cytoplasm and Golgi apparatus, consistent with its predicted function in glycosylation and growth regulation (Marsico et al., 2018). Together, these findings highlight CSGALNACT1 as a key regulator of tumor cell proliferation and a potential driver of malignant phenotypes (Shimbo et al., 2017).

In conclusion, we identify CSGALNACT1 as an actionable molecular node with potential therapeutic relevance in PTC. Its epithelial specificity may reduce systemic toxicity in targeted interventions. While our findings provide new insights into the molecular pathology of PTC, further studies are warranted to elucidate the mechanistic links between CSGALNACT1, tumorigenesis, and immune evasion, and to explore its value in therapeutic stratification.

## 5 Conclusion

This study identifies CSGALNACT1 as an epithelial-specific gene with independent prognostic significance in papillary thyroid carcinoma. Single-cell transcriptomic analyses reveal its alignment with epithelial differentiation trajectories, while functional assays demonstrate its role in promoting tumour cell proliferation. These findings position CSGALNACT1 as a potential biomarker and therapeutic target in thyroid cancer.

## Data Availability

Publicly available datasets were analyzed in this study. This data can be found here: The datasets presented in this study are available in the GEO (Gene Expression Omnibus) online repository with accession numbers GSE191288 and GSE193581 (https://www.ncbi.nlm.nih.gov/). Bulk-RNA data for thyroid cancer are from the TCGA project (https://portal.gdc.cancer.gov/, accessed on 9 February 2025). All public data are fully described in the Methods section. In addition, all data generated or analyzed during this study are included in this article.
